# Modeling Maintenance of Long-Term Potentiation in Clustered Synapses: Long-Term Memory without Bistability

**DOI:** 10.1155/2015/185410

**Published:** 2015-04-05

**Authors:** Paul Smolen

**Affiliations:** Laboratory of Origin, Department of Neurobiology and Anatomy, W. M. Keck Center for the Neurobiology of Learning and Memory, The University of Texas Medical School at Houston, Houston, TX 77030, USA

## Abstract

Memories are stored, at least partly, as patterns of strong synapses. Given molecular turnover, how can synapses maintain strong for the years that memories can persist? Some models postulate that biochemical bistability maintains strong synapses. However, bistability should give a bimodal distribution of synaptic strength or weight, whereas current data show unimodal distributions for weights and for a correlated variable, dendritic spine volume. Thus it is important for models to simulate both unimodal distributions and long-term memory persistence. Here a model is developed that connects ongoing, competing processes of synaptic growth and weakening to stochastic processes of receptor insertion and removal in dendritic spines. The model simulates long-term (>1 yr) persistence of groups of strong synapses. A unimodal weight distribution results. For stability of this distribution it proved essential to incorporate resource competition between synapses organized into small clusters. With competition, these clusters are stable for years. These simulations concur with recent data to support the “clustered plasticity hypothesis” which suggests clusters, rather than single synaptic contacts, may be a fundamental unit for storage of long-term memory. The model makes empirical predictions and may provide a framework to investigate mechanisms maintaining the balance between synaptic plasticity and stability of memory.

## 1. Introduction

A central question in neuroscience is the mechanism by which memories can be preserved for years. Long-term memories are at least in part encoded as specific patterns, or “engrams,” of strengthened synapses [1, 2], and long-term synaptic potentiation (LTP) persists for months* in vivo* [3]. How can specific groups of synapses remain strong for months or years despite turnover of macromolecules and fluctuations in the size and shape of synaptic structures?

Numerous mathematical models have been developed that describe maintenance of long-term memory (LTM) as dependent on bistability of synaptic weights, mediated by positive feedback loops of biochemical reactions, typically thought of as operative in dendritic spines. Proposed feedback mechanisms have relied on self-sustaining autophosphorylation of CaM kinase II [4, 5], persistent phosphorylation of AMPA receptors by protein kinase A [6], enhanced translation of protein kinase M *ζ* [7], or self-sustaining clustering of a translation activator, cytoplasmic polyadenylation element binding protein [8]. With these models, LTP switches a synapse from a state of low basal weight to a high weight state and turns on the positive feedback loop. The loop then operates autonomously to keep the synapse in the high weight state indefinitely. However, despite extensive investigation, empirical evidence of a bistable distribution of two distinct synaptic weight states has not, in fact, been obtained. Although some studies [9, 10] have suggested two distinct strength states for synapses, as measured by the amplitude of excitatory postsynaptic currents before and after a stimulus protocol, these studies have only examined the early phase of LTP (<1 h), which does not depend on protein synthesis or other processes necessary for long-term memory storage. Therefore, those data do not address the dynamics of long-term memory storage. In addition, a demonstration of synaptic bistability would require not only finding two distinct synaptic strength states but also finding that a set of different protocols for LTP induction (e.g., different patterns of stimuli, or localized application of pharmacological agents) commonly switched synaptic weights between the* same* two stable states. Such a demonstration has not been attempted. In addition, modeling suggests that stochastic fluctuations of macromolecule numbers within a small volume such as a spine head are likely to destabilize steady states of biochemical positive feedback loops, causing randomly timed state switches ([11]; see [5] for an opposing view). Finally, in hippocampal neuron cultures, continuous and extensive fluctuations of postsynaptic density (PSD) morphology are observed and are driven in part by synaptic activity [12]. Such dynamics would seem difficult to reconcile with only two, or a few, stable weight states.

Empirical distributions of the weights of excitatory synapses onto cortical or hippocampal pyramidal neurons appear unimodal (a single peak) rather than bimodal and are commonly heavy-tailed (skewed towards high weights) [13–18]. Some histograms are based on relatively small numbers of measurements, so that some bimodality might be present but hidden in variability among bins. However, the weight distribution of Song et al. [18] is based on measurements of several hundred excitatory postsynaptic potential (EPSP) amplitudes and appears to particularly disfavor the bimodal hypothesis. A large number of measurements are fit well by a log-normal distribution (i.e., a normal distribution with the logarithm of the volume on the *x*-axis).

In addition, a histogram of the volume of dendritic spines, based on a large number of individual measurements (~10,000) in mouse auditory cortex, is clearly unimodal, heavy-tailed, and approximately log-normal [19]. Observations support a substantial correlation between spine volume and synaptic weight. Spine volume is approximately proportional to the postsynaptic density size [20–22] and to the number of postsynaptic AMPA receptors [22] as well as to the amplitude of the EPSC measured following localized glutamate uncaging [23]. Thus it is plausible, and we assume that increases/decreases in spine volume can serve as a proxy for LTP/LTD.

If synaptic weights and correlated spine volumes are not in fact bistable, how can patterns of strong synapses be maintained for very long times? Two observations support a mechanism based on metastability of small clusters of large dendritic spines, corresponding to groups of strong synaptic contacts. The first observation is that although spine volumes fluctuate, some large spines are extremely stable, existing for months (in sensory cortex [24] or in motor cortex [25]). The second is that induction of late, protein synthesis-dependent LTP (L-LTP) at a spine facilitates L-LTP expression at nearby spines, on the same dendritic branch, that coincidentally receive stimuli too weak to support L-LTP if given alone [26]. This observation supports the “clustered plasticity hypothesis” in which clusters of spines on a single dendritic branch, rather than single spines, may serve as “primary functional units” for storage of LTM [27]. This hypothesis is now supported by substantial recent data [28]. For example (1) in motor cortex, learning induces coordinated formation of small spine clusters on a given dendritic branch [29] (2) morphologically, spines are grouped into small clusters on pyramidal dendrites [30] and (3) in rat hippocampal slice cultures, spontaneous coactivation of dendritic spines is frequent and is clustered, occurring more often for spines within 8 *μ*m of each other [31].

Here an initial, relatively phenomenological model is developed describing synaptic weight changes due to competing processes of LTP (corresponding to spine growth) and long-term synaptic depression (LTD) (corresponding to spine shrinkage). Assuming volume changes are a proxy for weight changes, weight changes are simulated for discrete intervals of 1 day, over times of months or years. The discrete intervals were chosen to simulate the dynamics observed in experiments where volumes are imaged at intervals of ~1 day [19, 32]. In the model, a single synapse corresponds to a dendritic spine. Daily growth of synapses or spines corresponds to LTP and daily shrinkage to LTD. Each day the magnitude of LTP is governed by a Gaussian random variable, as is that of LTD (Methods). These magnitudes are also approximately proportional to the preexisting weight. As suggested by recent data ([24, 25, 32] but see [19]), a volatility factor was introduced so that the weights of larger synapses fluctuate less. This factor proved necessary for large synapses to remain stable for months (see Discussion).

Model parameter sensitivity was lessened when synapses were modeled as coupled into small clusters (~10 spines, modeled as on the same dendritic branch and able to interact). In accordance with data [32, 33], the model also incorporates disappearance or silencing of small synapses and compensatory regeneration of new synapses. When the dynamics of 1,000 small clusters were simulated, the weight distribution of the entire ensemble of individual synapses converged to a steady-state, log-normal form. Individual clusters remained stable for many years, with the average number of active synapses maintained in a range consistent with empirical data. The magnitude of daily changes in synaptic weight approximated a normal distribution except for an extra peak at Δ*W* = 0, which constitutes a model prediction.

Persistence of imposed memories for years was also simulated. If a subset of synapses was reinitialized to have large weights, this subset maintained large average weights for ~2 yrs, corresponding to persistence of a pattern of strong synapses that might serve as the engram for a memory. Some memories however persist for even longer times. In support of the clustered plasticity hypothesis, our simulations suggest that such memories might be encoded as a pattern of specific, stable clusters of active synapses. In simulations, such a pattern remained stable for many years.

## 2. Methods

Weight evolution in 1,000 independent clusters was simulated. Each cluster consists of *N*_cl⁡_ synapses, corresponding to *N*_cl⁡_ individual spines. At a given time, most of these synapses are “active,” with synaptic weight *W* ranging from ~0.2 to 10. The remaining synapses are “silent,” with a very low, basal weight of 0.05. Weight evolution is simulated using discrete, large time steps Δ*t*, considered to correspond to 24 h. At each time step, all weights are synchronously updated. The size and direction of a weight update at a given synapse are assumed uncorrelated with that at a neighboring synapse and with the preceding update at the given synapse. These assumptions are supported by data describing spine volume changes on pyramidal dendrites [32].

**Figure 1:**
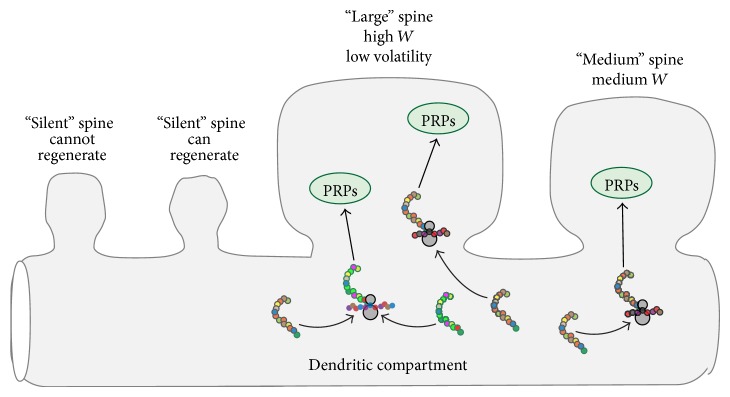
Schematic illustrating key model elements. Two silent synapses, corresponding to two small dendritic spines, are adjacent to two active synapses corresponding to larger spines. Only the silent synapse next to an active synapse is currently eligible for regeneration. Although the dynamics of mRNAs or locally synthesized proteins are not simulated, large synapses are implicitly assumed to capture more of these resources. This is illustrated by a greater flow of mRNAs (colored strands) onto ribosomes near the largest synapse and a greater flow of plasticity-related proteins (PRPs) into that synapse. This resource capture attenuates the amount of resource available to support growth of all synapses in the cluster. The average amplitude of LTP events is thereby reduced when more synapses in a given cluster are strong.

Figure 1 schematizes key elements of the model. A cluster of four spines is illustrated; two are active. High values of *W* correspond to large spines. Very small spines correspond to “silent” synapses. For each active synapse, two independent processes change synaptic weights during each time step. An “LTP” process increases *W* and an “LTD” process decreases *W*. Strong synapses consume more resources for maintenance, corresponding to locally available mRNAs/proteins. The model thus assumes that the more strong synapses present in a cluster, the fewer resources are available to support synaptic growth (LTP). Thus the amplitude of LTP decreases with the number of strong spines. Empirically, smaller spines are more volatile. Thus, the model assumes that the amplitude of LTP is also proportional to a volatility factor that decreases as *W* increases (1)-(2) as is that of LTD (3). LTP and LTD add to give the net change in *W* per time step (4).

**Figure 2:**
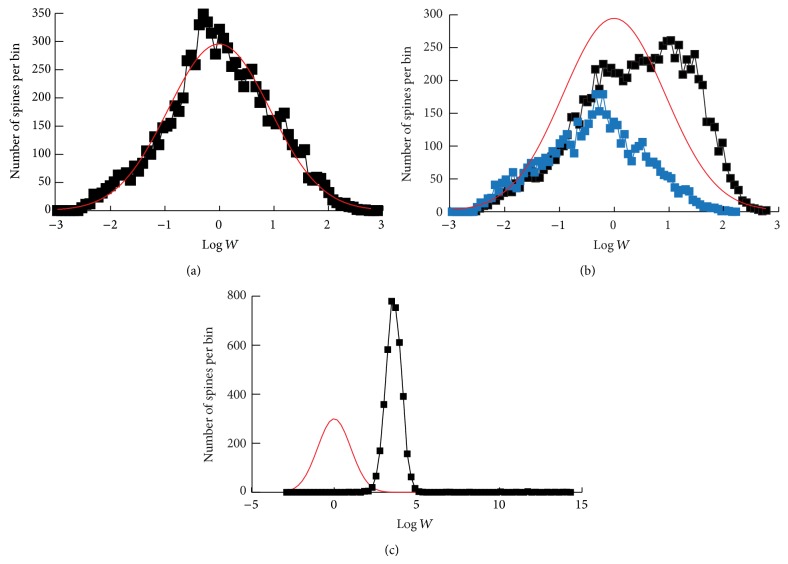
Simulated distributions of synaptic weights. (a) Distribution for 1,000 independent clusters with *N*_cl⁡_ = 10. Black trace, histogram with 80 bins illustrating an approximately log-normal distribution of the 10,000 weights. Each bin is equal in width in natural log units. Red curve, a log-normal distribution (mean at 0.0131, standard deviation of 0.9341), fitted by MATLAB, that approximately reproduces the histogram. The histogram was constructed after 50,000 simulated days to ensure a steady state. (b) Black and red traces, similar to (a), except the mean and standard deviation of LTP, parameters *a*_1_ and sd_1_, are fixed at 0.16 and 0.04, respectively. Blue trace, the histogram of *W* is shifted to much lower values when *a*_1_ is decreased by 2%. (c) Weight dynamics without regeneration of synapses. The histogram of the active synapses shifts to much greater values (black trace). ~45% of synapses are silent, unable to regenerate, and not included in the histogram. This histogram is an approximate steady state, although with no regeneration, all synapses would become silent after a much longer time. Red curve, normal distribution from (a).

For each time step, the LTP and LTD amplitudes are proportional, respectively, to Gaussian random variables *r*_1_ and *r*_2_. These variables have respective means *a*_1_ and *a*_2_, and sd_1_ and sd_2_ are the standard deviations. *a*_1_ and *a*_2_ are substantially larger, by a fixed factor of 4, than are sd_1_ and sd_2_. Thus *r*_1_ and *r*_2_ are very rarely negative, but if either becomes negative it is reset to zero. A synapse is “strong” if its weight is above a threshold *T*_st_. The average LTP amplitude *a*_1_ is a decreasing function of the number of strong synapses in a given cluster, denoted as *N*_st_. With *N*_cl⁡_ the total number of synapses in a cluster, the average LTP amplitude *a*_1_ decreases linearly with *N*_st_, from a maximum amplitude *x*_2_ (for *N*_st_ = 0) to a minimum *x*_1_ (for *N*_st_ = *N*_cl⁡_). For the simulation of Figure 2(b) with this amplitude decrease removed, *a*_1_ and thus sd_1_ are fixed parameters. For comparison, simulations were also carried out in which *r*_1_ and *r*_2_ were drawn from exponential distributions. With exponential distributions *r*_1_ and *r*_2_ are always nonnegative, with probability densities that peak at 0 and decay exponentially for increasing positive values. The corresponding decay rate constants were varied independently within the range [0.5,3.0].

LTP and LTD are also proportional to a volatility factor VO_*W*_, decreasing with *W*:

(1) $$\begin{array}{c} \text{V}{\text{O}}_{W}=\left\{v_{\text{hi}}-\left(v_{\text{hi}}-v_{\text{lo}}\right)\left(\frac{W}{W+W_{\text{med}}}\right)\right\}. \end{array}$$

From (1), VO_*W*_ decreases from the parameter *v*_hi_ (for *W* = 0) to *v*_lo_ (for *W* ≫ *W*_med_). When *W* = *W*_med_, VO_*W*_ is midway between *v*_hi_ and *v*_lo_.

LTP and LTD amplitudes are also multiplied by the preexisting value of *W*. To keep *W* bounded the LTP amplitude is also multiplied by a decreasing function of *W* that has two parameters, *k*_hi_ and *W*_hi_. Combining factors the LTP amplitude is

(2) $$\begin{array}{c} A_{\text{LTP}}=W\cdot r_{1}\cdot \text{V}{\text{O}}_{W}\left[1-k_{\text{hi}}\left(\frac{W}{W+W_{\text{hi}}}\right)\right]. \end{array}$$

The LTD amplitude is

(3) $$\begin{array}{c} A_{\text{LTD}}=W\cdot r_{2}\cdot \text{V}{\text{O}}_{W}. \end{array}$$

At each time step, for each active synapse,

(4) $$\begin{array}{c} W_{\text{new}}=W_{\text{old}}+A_{\text{LTP}}-A_{\text{LTD}}. \end{array}$$

If *W* falls below a threshold *T*_wk_, the synapse is reset to be silent. For each silent synapse at each time step, the probability for regeneration *P*_ACT_ increases with the number of strong synapses in its cluster, to a maximal value *P*_bas_:

(5) $$\begin{array}{c} P_{\text{ACT}}=P_{\text{bas}}\frac{N_{\text{st}}}{N_{\mathrm{cl}}}. \end{array}$$

In addition, regeneration only occurs if an adjacent synapse is strong. In a cluster, synapse 1 can only switch to active if synapse 2 is strong, and synapse 5 can only switch if synapse 4 and/or 6 is strong. A switch resets *W* to *W*_reset_, above *T*_wk_.

For all simulations, to ensure initial weight, distributions were at steady state and distributions and other quantities were computed only after 50,000 simulated days. *W* is nondimensional and *t* has units of hrs.

It is necessary that simulated time steps plausibly correspond to the common empirical interval of 1 day between spine imaging sessions. Therefore, it was necessary to scale LTP and LTD amplitudes so that for a time step the simulated per cent change in *W*, averaged over all synapses, agreed with an average daily empirical change in *W*. Empirically Yasumatsu et al. [32] presented a piecewise-linear relationship between spine volume *V* and its change Δ*V* (Figure  7(b) of [32]) under control conditions (normal synaptic activity). This model, which they denote C-1, was also able to predict an empirical steady-state spine volume distribution (Figure  8(e) of [32]). Model C-1 is

(6) ΔV=−0.16·V+0.01 for  V≤0.25 μM3  (most  spines),ΔV=0.12·V−0.06 for  0.25 μM3<V≤0.5 μM3,ΔV=0 for  V>0.5 μM3.

**Figure 3:**
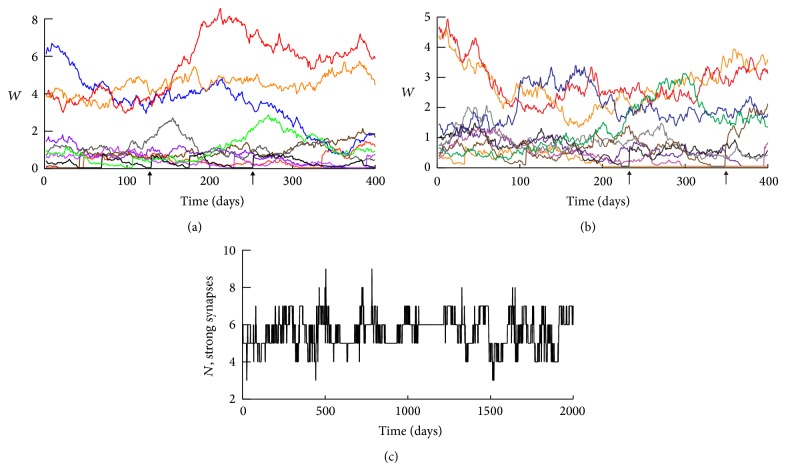
Dynamics of synaptic weights. (a) and (b) Representative time courses for 10-synapse clusters over 400 days. Synapses with higher *W* exhibit less volatility (smaller percent changes in *W*). Synapses with high weights often remain strong for >1 yr. (c) A representative time course of the number of strong synapses, *N*_st_, in a 10-synapse cluster. Although individual weights undergo large fluctuations, *N*_st_ is extremely stable, remaining between 4 and 7 for periods of over a year and rarely leaving this range during 5.5 years.

**Figure 4:**
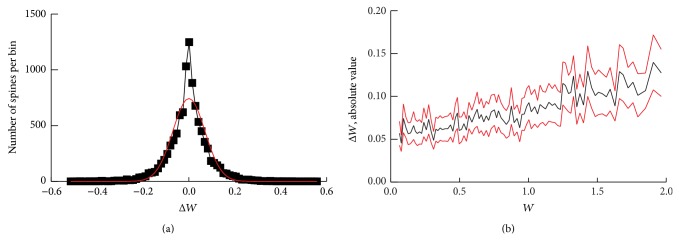
Distributions of the magnitude of synaptic weight change. (a) Black trace, histogram with 80 bins illustrating the steady-state distribution of weight update amplitudes for the active synapses in Figure 2(a). These amplitudes consist of all the synchronous weight updates for the 9,482 active synapses (out of 10,000 total), at a given time step. Red curve, a normal distribution (mean 0.0, standard deviation 0.07) that approximates the histogram excepting the sharp peak for update amplitudes near zero. (b) A histogram of Δ*W versus W* shows a slightly increasing, relatively linear trend. 80 bins for *W* are equally spaced on a log scale. Black trace, mean values of Δ*W*. Red traces, mean ± 1 standard deviation.

**Figure 5:**
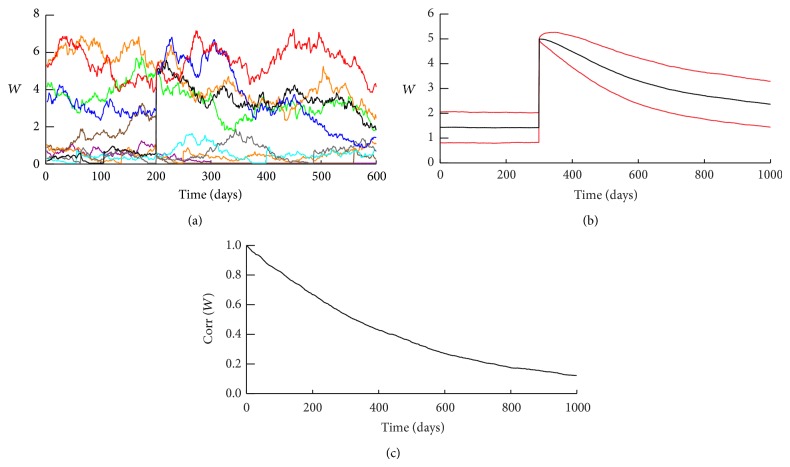
Weight dynamics with imposed LTP of a subset of synapses. (a) A representative time course of a 10-synapse cluster. *t* = 0 corresponds to the distribution of Figure 2(a). At *t* = 200 days, *W* was set to a high value of 5 for synapses 1–5 and a low value of 0.5 for synapses 6–10. Over 400 days the distinction between strong and weak synapses was largely preserved, with synapses 1–4 remaining at high weights. (b) Dynamics of the potentiated synapses in 10,000 10-synapse clusters. For each cluster, synapses 1–5 were potentiated as in (a); thus dynamics of 5000 synapses are illustrated. Black trace, time course of the average weight of these synapses. Red traces, ±1 standard deviation from average. 700 days after LTP, the average weight remains >1 standard deviation above the steady-state average. (c) Correlation coefficient describing the evolution of synaptic weights during maintenance of the steady-state distribution. For 10,000 synapses in 10-synapse clusters, in the distribution of Figure 2(a), the Pearson correlation coefficient *R* was calculated between the values of *W* at *t* = 0 and the values at later times.

From this relationship, combined with the plotted volumes in Figures  1(b) and  1(c) of [32], it can be inferred that the average percent daily change in *V* lies within the range of ~14–20%. One cannot infer a more precise value from these data. For comparison, in the model's steady-state weight distribution of Figure 2(a), the percent change in *W* during a time step, averaged over all 10,000 synapses, is 16.5%. Assuming *V* is a proxy for *W*, this qualitative agreement between simulated and empirical average weight changes suggests that the fixed time step in Figures 2–5, which is otherwise arbitrary, can be taken to correspond to approximately 1 day of weight dynamics.

Simulation of the Pearson correlation coefficient *R* was done as follows. Let *X*_*i*_ denote the total set of *n* synaptic weights at a reference time, with *i* the indexing variable from 1 to *n*. For 1,000 10-synapse clusters, *n* = 10, 000. Let *Y*_*i*_ denote the set of *n* weights at any later time step. As *t* increases from the reference time, *Y*_*i*_ will evolve and *R* will decline from 1. Let $\overset{-}{X}$, $\overset{-}{Y}$ denote the means of *X*_*i*_, *Y*_*i*_. The standard equation was used:

(7) $$\begin{array}{c} R=\frac{\sum_{i=1}^{n}\left[\left(X_{i}-\overset{-}{X}\right)\left(Y_{i}-\overset{-}{Y}\right)\right]}{\sqrt{\sum_{i=1}^{n}{\left(X_{i}-\overset{-}{X}\right)}^{2}}\sqrt{\sum_{i=1}^{n}{\left(Y_{i}-\overset{-}{Y}\right)}^{2}}}. \end{array}$$

Standard parameter values, used in all simulations unless stated otherwise, are as follows:

(8) $$\begin{array}{c} N_{\mathrm{cl}}=10,\;\;W_{\text{reset}}=0.4,\;\;T_{\text{wk}}=0.08,\\ T_{\text{st}}=0.8,\;\;v_{\text{hi}}=4.0,\;\;v_{\text{lo}}=0.2,\\ W_{\text{med}}=0.4,\;\;x_{1}=0.144,\;\;x_{2}=0.18,\\ a_{2}=0.16,\;\;k_{\text{hi}}=0.05,\;\;W_{\text{hi}}=20.0,\;\;P_{\text{bas}}=0.1. \end{array}$$

In Supplementary Material (available online at http://dx.doi.org/10.1155/2015/185410), a Java program is given that executes simulations from Figures 2–4.

## 3. Results

Figure 2(a) illustrates the approximately log-normal distribution of synaptic weights obtained at steady state. Here, 1,000 clusters of synapses were simulated, with *N*_cl⁡_ = 10. The black trace is the resulting histogram of synaptic weight *W*; the red trace is a log-normal distribution fitted to the histogram. There are 10,000 values of *W* in the histogram. The range of *W* spans approximately 5 natural log units (i.e., a multiplicative factor of ~150). This range is similar to data describing the range of dendritic spine volumes [19, 32]. In this steady state, the relative synaptic weight change per time step, averaged over all synapses, is 16.5%. This percent is in qualitative agreement with data ([32]; details in Methods). This agreement is necessary for the model time step to represent the empirical interval of 1 day between imaging sessions.

To prevent this distribution from being overly sensitive to the average daily LTP amplitude, the model assumes this amplitude decreases with the number of strong synapses in the cluster to which the synapse belongs. Starting from Figure 2(a), when the mean LTP amplitude was decreased by 5%, the mean of *W* decreased by only 16%. In contrast, Figure 2(b) illustrates that in a model variant with mean and standard deviation of the LTP amplitude fixed, the weight histogram was shifted to the right of the log-normal distribution from Figure 2(a) and has a shape clearly distorted from log-normal with a much steeper cutoff at high *W*. To attempt to improve the histogram, the mean LTP amplitude was decreased by 2%. This small change resulted in a large shift of the histogram to the left (blue trace), and 56% of the synapses became silent (not included in the histogram). A similar distribution, with extreme sensitivity to mean LTP amplitude, resulted from a model variant in which synaptic clustering was deleted by fixing the mean and standard deviation of the LTP amplitude and also fixing the synapse regeneration probability *P*_ACT_ (5). These model variants with extreme sensitivity were not analyzed further.

Removal of synaptic regeneration greatly alters the distribution and dynamics of synaptic weights. The distribution (Figure 2(c)) no longer resembles data. The distribution is strongly bimodal, with almost 50% of synapses silenced at the low basal weight, unable to regenerate, and the remainder in a narrow distribution centered at very high weights.

One other model variant was also simulated, in which the Gaussian random variables *r*_1_ and *r*_2_ that govern the amplitudes of individual LTP and LTD increments were replaced by random variables drawn from exponential distributions (Methods). However, these simulations failed to produce synaptic weight distributions that resembled experimental data. If the decay rate constants for the two exponential distributions differed by more than twofold, almost all synaptic weights either ran to infinity (if the mean LTP amplitude was greater) or converged to a low basal synaptic weight imposed as a floor in the model (if the mean LTD amplitude was greater). If the rate constants were similar a positive synaptic weight distribution could be obtained, but the shape of this distribution was itself exponential. It was not close to log-normal and did not resemble experimental distributions. Therefore this model variant was also not analyzed further. All following results are therefore based on the first model variant described above, that of Figure 2(a), with synaptic regeneration and an LTP amplitude that decreases with the number of strong synapses in a cluster. Equations and parameter values are in Methods.

Typical time courses for clusters with regeneration are illustrated in Figures 3(a) and 3(b). Strong synapses are much more stable on average, in agreement with data demonstrating that large dendritic spines are more persistent [32, 34, 35]. Strong synapses often maintain high values of *W* for a year or more, whereas weak synapses show much larger relative (percent) fluctuations in *W*. Weak synapses often drop to a very low basal weight. These “silent” synapses can regenerate, evident as vertical jumps in time courses near the bottom of Figures 3(a)-3(b) (e.g., arrowheads below *x*-axes).

Figure 3(c) illustrates a typical time course for the number of strong synapses *N*_st_ in a cluster (with weights greater than a threshold *T*_st_ = 0.8). Clusters are very stable in that, for *N*_cl⁡_ = 10 synapses per cluster, *N*_st_ almost always remains between 4 and 7 for years. Simulations with *N*_cl⁡_ = 15 or 20 also had very stable clusters. However, for a smaller *N*_cl⁡_ = 5, the model no longer simulated a log-normal synaptic weight distribution at steady state. A bimodal distribution occurred, with synapses either at basal weight or very strong.

Minerbi et al. [36] recorded synaptic dynamics for many days in cortical neuron cultures. Rather than spine volume, they examined variations in the size of PSD-95:GFP puncta, that is, localized concentrations, with a size corresponding to spines, of the postsynaptic density protein PSD-95 fused to a GFP fluorophore. Their dynamics are qualitatively consistent with those illustrated in Figures 2(a) and 3 in that (1) new synapses were continually formed, (2) synapses whose size was reduced beneath some threshold, analogous to the model threshold *T*_wk_, were eliminated, and (3) large synapses tended to shrink and small synapses to grow. In accordance with (3), stability of a simulated steady state, unimodal distribution such as that of Figure 2(a) requires that individual synapses with weights above the peak of the distribution decrease their weight on average and* vice versa* for those with low weights. Empirically Matsuzaki et al. [37] found that smaller spines are more likely to undergo stable enlargement in response to an LTP induction protocol.

Loewenstein et al. [19] and Yasumatsu et al. [32] present plots of daily changes in spine volume* versus* initial volume.  Figure 4(a) illustrates a simulated histogram of the amplitudes of the daily changes in synaptic weight, Δ*W*, at steady state. The majority of the histogram is fitted well by a normal distribution with the clear exception of the narrow peak close to Δ*W* = 0. A scatter plot of Δ*W versus W* revealed that over almost the entire range of *W*, the amplitude of Δ*W* varied from near zero to a peak value of ~0.3–0.5. Therefore, the peak near zero is not due exclusively to large synapses.  Figure 4(b) plots daily changes in weight* versus* initial weight. The plot is qualitatively linear, which is not surprising because, in the model, the average daily LTD and LTP amplitudes contain terms proportional to *W* (Methods, (2), (3)). However, the plot further illustrates that the average change in *W* varies much less than does *W* itself. As *W* increases from 0.05 to 2.0, the absolute value of Δ*W* increases only from ~0.05 to 0.12. Thus, the relative change in *W* (i.e., Δ*W*/*W*) decreases substantially as *W* increases. This prediction of the model appears in accordance with the data from Yasumatsu et al. [32] but not with the data of Loewenstein et al. [19] for which this relative change appears nearly constant (see Discussion).

Strong synapses can maintain high weights for many months in simulations. However, what if the weight distribution is altered by an imposed large perturbation that sets high weights for a specified subset of synapses? Such a perturbation might correspond to formation of a specific, long-term memory engram. Will the subset of strong synaptic weights remain elevated for months, corresponding to long-lasting storage of a memory? Starting from the distribution of Figure 2(a), for all of the 1000 10-synapse clusters, synapses 1–5 were reset to a high weight of 5.0 at *t* = 200 days. This weight is well above the steady-state mean *W*. The other 5 synapses were reset to a low weight (0.5). We then simulated the dynamics of all clusters for a further 700 days.  Figure 5(a) illustrates a typical time course of weights for a cluster. After the reset, the weights fluctuate but 400 days later, all but one of the strong synapses have maintained *W* above the steady-state average.  Figure 5(b) illustrates the time course of resetting and decay for all 5,000 synapses that were reset to *W* = 5.0. Their average weight decays slowly such that 700 days after reset this average is still about twice the steady-state average of *W*. The lower red trace, one standard deviation below the average weight, is also still above the steady-state average.

If the steady-state distribution of *W* is evolved without any perturbation, the time scale for decorrelation of synaptic weights from their specific values at a given time is, perhaps surprisingly, quite long, similar to the time scale for the decay of perturbed synaptic weights. Starting from the distribution of Figure 2(a), the Pearson correlation coefficient between the weights of the 10,000 synapses decays with a time constant of approximately 500 days (Figure 5(c)), similar to the dynamics of Figure 5(b).

## 4. Discussion

With simulations of isolated, uncoupled synapses (Figure 2(b)), weight distributions were extremely sensitive to model parameters. This model variant was therefore not a plausible description of synaptic dynamics, because biophysical parameters are expected to vary somewhat between spines, dendritic branches, and individual neurons. This sensitivity was eliminated when synapses were modeled as coupled into clusters (~10 synapses). In the model a single “synapse” corresponds to a dendritic spine. Yadav et al. [30] have reported similar spine clusters on primate cortical pyramidal neurons. On a scale of ~4–10 *μ*m along dendritic branches, numerous clusters of 5–15 spines were identified algorithmically. This distance scale and cluster number appears to correspond to the observations of Takahashi et al. [31] that spontaneous coactivation of dendritic spines is clustered, occurring more often for spines within 8 *μ*m of each other. We do note recent analysis by a different group [38] failed to support clustering of this type and scale.

With coupling into clusters of ~10 synapses, when the dynamics of 1,000 clusters were simulated, the weight distribution of individual synapses converged to a stable steady-state, log-normal form (Figure 2(a)). Individual synaptic weights fluctuate but the distribution is stable indefinitely.

Data illustrates that spines compete for LTP expression; that is, local resources (possibly amounts of key proteins) are limited such that the amount of LTP at a given spine decreases if LTP is simultaneously induced at multiple spines close together on the same dendritic branch [26]. Our mechanism of coupling synapses into clusters is similar in that it corresponds to an additional form of resource competition. However, in the model, competition is generated by ongoing maintenance of multiple synapses rather than only by simultaneous LTP of multiple synapses. Thus, the mean magnitude of LTP at a given synapse during a simulated day was assumed to be a decreasing function of the number of other large synapses in the same cluster (Methods). Ongoing maintenance of large spines is assumed to consume resources (proteins, RNA) that would otherwise be available for strengthening of neighboring spines, so that their mean LTP magnitude decreased. Current data does not support or refute this coupling mechanism. However, it appears plausible and constitutes a model prediction. The competition described by Govindarajan et al. [26] occurs over a distance scale of 10–20 *μ*m, comparable to, although slightly larger than, the scale of 5–10 *μ*m posited by Yadav et al. [30] for clusters of ~5–10 spines. Govindarajan et al. [26] did not determine the rate at which competition falls off with distance. However, they did determine this rate for another measure of synaptic cross talk, the ability of a weakly stimulated spine to capture plasticity-related factors synthesized in response to strong stimulation of a nearby spine (synaptic tagging and capture, resulting in LTP at the weakly stimulated spine). LTP was observed for distances up to ~50 *μ*m. These data suggest that, for clusters of ~10 spines as simulated here, distance between spines might not limit the efficacy of resource competition. However, these data do not address whether competition could occur over a longer distance scale, for example, 50 *μ*m. To assess this question groups of spines spaced along a dendritic branch would need to be concurrently stimulated, and LTP quantified.

To simulate a distribution spanning a broad range of synaptic weights, as is observed empirically, it was critical to model synaptic regeneration. To obtain distributions similar to that of Figure 2(a), synapses that fell to a low basal weight needed to have, on successive days, a probability of weight reset to a higher, “active” value. This method of simulating regeneration was chosen to maintain equal average numbers of synaptic loss and regeneration events within any given cluster. Empirically, the number of active spines in a cluster is relatively low (~3–7) [30]. Thus for clusters with low numbers of active spines to serve as functional memory storage units, as suggested by the clustered plasticity hypothesis [27], the average cluster size would need to be stable to avoid disappearance of clusters. An important question in neuroscience is how the observed regeneration of spines avoids disrupting stored memories or forming spurious “memories.” It is plausible that new, or regenerated, spines are generically not strong (as in our model) and therefore cannot participate in memory storage unless they are potentiated by stimuli that induce long-term memory. However, further empirical investigation of this question, in conjunction with modeling, is needed.

The model of Loewenstein et al. [19] simulates daily changes in the volume of dendritic spines and obtains a steady-state log-normal distribution of spine volumes very similar to the empirical distribution found by these authors. These important results have clarified the necessity of reconciling unimodal weight distributions with stable storage of long-term memory. Their model consists of an Ornstein-Uhlenbeck stochastic process in which the logarithm of the volume of any given spine is directly incremented each day. The model presented here may constitute a further advance, in that it represents more biophysical elements, such as synapse loss/regeneration and synapse clustering. The LTP and LTD processes in the model (1) act directly on the synaptic weight rather than on its logarithm and (2) can be thought of as due to a large number of individual biochemical events occurring during a simulated day and corresponding to insertion and removal of individual molecular complexes or slots. Point (2) connects the current model with the more detailed molecular model of Lisman and Raghavachari [39], in which LTP and LTD correspond, respectively, to insertion and removal of “slot” complexes, each consisting of a small number of AMPA receptors together with associated scaffolding proteins and, possibly, kinases or other signaling proteins. Additional elements of our model that may be consistent with that of Lisman and Raghavachari [39] are as follows. If over a day, the time intervals between individual insertions of “slot” complexes as well as the intervals between removals of complexes follow Poisson distributions, and if there are on average more than ~20 insertions and removals/day, then by the Central Limit Theorem the sums of these Poisson processes will approximate Gaussian random variables. These Gaussian variables would correspond to the model's Gaussian random variables *r*_1_ and *r*_2_, to which the daily LTP and LTD amplitudes are proportional (2), (3). Similarly, if the individual time intervals were Gaussian random variables, their sum would be Gaussian. It also appears plausible that average numbers of slot insertions and removals are proportional to the preexisting size of a spine, corresponding to the model assumption that mean LTP and LTD amplitudes are approximately proportional to synaptic weight.

Recent data [40] are consistent with these ideas and also suggest such slot complexes include presynaptic components and release sites. Current data has not directly examined whether there is spatial clustering of presynaptic boutons at the distance scale of ~5 *μ*m that corresponds to putative postsynaptic spine clusters. However, at this distance scale, such clusters would correspond to a single axon and spines in a cluster would thus be coactivated by a common input, consistent with the hypothesis that such clusters constitute functional units in the formation and storage of specific memories.

In the model, the average relative (percent) change in *W* over a day decreases substantially as *W* increases (Figure 4(b)). This result is essential for the model to represent stable long-term memory storage, because selective stability of strong synapses is only found if the relative change in *W* decreases in this manner. Are these dynamics supported by current data? Relevant data describes the relationship of spine volume changes Δ*V* to *V*. These data are, however, contradictory. Loewenstein et al. [19] illustrate a substantially larger relative variation in Δ*V* (Figure 4(c) of [19]), such that Δ*V* and *V* appear approximately proportional. However, Yasumatsu et al. [32] show a different relationship, for which smaller spines have an average Δ*V* similar to large spines (Figure 1(c) of [32]). The latter relationship, but not the former, appears compatible with our assumption. It is plausible that the difference in synaptic dynamics in these studies was generated, at least in part, by substantial differences in experimental conditions. Yasumatsu et al. [32] imaged CA1 pyramidal neurons in cultured rat hippocampal slices at postnatal days 17–23, whereas Loewenstein et al. [19] did craniotomies to allow* in vivo* imaging of dendrites in auditory cortex of mice approximately 6 months old. In addition, filopodia (protrusions without spine heads or with very small heads) were eliminated from the former study, but not the latter. Clearly further empirical investigation is needed to clarify these critical aspects of synaptic dynamics.

At steady state the magnitudes of the daily changes in weight were distributed approximately normally except for an extra peak centered at Δ*W* = 0 (Figure 4(a)). Empirical data has not reported such a peak close to zero [19]. Therefore, this peak could constitute a deficiency of the model. However, data does indicate that a minor percentage of spines are stable for months or years [24, 25]. These data suggest that a subpopulation of spines with very small daily weight changes might exist, but not yet be resolved due to empirical sensitivity limits. The current model does not represent the biochemical processes that might underlie long-term stability of such a subpopulation. However, recent studies support a relevant hypothesis that ongoing spontaneous neuronal activity is critical for long-term maintenance of synaptic strength. Models have suggested that such activity can preferentially maintain synapses that are already strong [41] possibly by preferentially reactivating stored patterns of strengthened synapses [42]. Empirically, a temporary induced knockdown of NMDA receptor function can irreversibly eliminate remote memories [43], plausibly by preventing spontaneous activity from potentiating and thereby maintaining synapses. Ongoing LTP increments that are necessary to maintain strong synapses, whether single or clustered (as in the current model), may correspond to frequent spontaneous or environmentally induced activity, which may induce repeated cycles of NMDA receptor-dependent LTP.

Simulations (Figures 5(a)-5(b)) illustrate that the model can store a specific memory trace, for ~1 yr, corresponding to persistence of a pattern of strong synapses. However, in humans, some memories persist for many years. In accordance with the hypothesis of Govindarajan et al. [27], such memories might be encoded at the cluster level rather than the single-synapse level, as a set of specific, stable clusters of active synapses. In simulations, these clusters were stable indefinitely (Figure 3(c)). They maintained a range of strong synapse numbers (~4–7) similar to the range suggested by data demonstrating clustering on neocortical pyramidal dendrites [30]. Because each cluster was stable indefinitely, if a pattern of such clusters was established, that pattern would persist.

In simulations, stable clusters were found when the total number of synapses in a cluster was 10 (Figure 3(c)), or 15–20. However, for five synapses per cluster, anomalous dynamics resulted. A bimodal synaptic weight distribution, not resembling empirical data, was seen at steady state. We do not take this result to be a prediction of a minimum empirical cluster size. Instead, we believe that the model should be improved in future work to avoid or reduce this dramatic change in dynamics.

The model makes additional predictions. When comparing spines of similar sizes in different clusters, the average volume change between imaging sessions is predicted to be less positive (or more negative) if other spines in a cluster are large. In addition, because synaptic weights and spine volumes are not considered bistable, different induction protocols for late, protein-synthesis dependent LTP are predicted to induce clearly different amplitudes of synaptic weight increase or of average spine volume increase. Without bistability, repeated applications of stimulus protocols should, at least in some cases, further enhance L-LTP.

## Supplementary Material

The supplementary Java program outputs multi-column ASCII files from which the plotted traces in Figs. 2A, 3A, 3C, 4A, and 4B can be reproduced. Because these simulations use random variables, the traces will not be reproduced exactly, but approximately. To run the simulations, save the Java program as file “figms.java”. Install Java using, for example, the Java Development Kit (JDK) from Oracle. Type “javac figms.java” to compile and “java figms” to run.
