# Supplementary material for: Modeling Maintenance of Long-Term Potentiation in Clustered Synapses: Long-Term Memory without Bistability
Source: Neural Plast. 2015 Apr 5;2015:185410. doi: 10.1155/2015/185410 (PMC4402204; doi:10.1155/2015/185410)
Supplement: Supplementary file 1 — The supplementary Java program outputs multi-column ASCII files from which the plotted traces in Figs. 2A, 3A, 3C, 4A, and 4B can be reproduced. Because these simulations use random variables, the traces will not be reproduced exactly, but approximately. To run the simulations, save the Java program as file “figms.java”. Install Java using, for example, the Java Development Kit (JDK) from Oracle. Type “javac figms.java” to compile and “java figms” to run. [file 185410.f1.rtf]

The Java program below outputs multi-column ASCII files from which the plotted traces in Figs. 2A, 3A, 3C, 4A, and 4B can be reproduced. Because these simulations use random variables, the traces will not be reproduced exactly, but approximately. To run this program, cut off this text header and save the Java program as file “figms.java”.  Install Java using, for example, the Java Development Kit (JDK) from Oracle. Type “javac figms.java” to compile and “java figms” to run.

import java.io.*;

public class figms

 {  public static void main (String args[]) throws IOException {

    PrintWriter out1 = new PrintWriter(new FileWriter("wdelw.txt"));
    PrintWriter out2 = new PrintWriter(new FileWriter("delhist.txt"));
    PrintWriter out3 = new PrintWriter(new FileWriter("deldist.txt"));
    PrintWriter out4 = new PrintWriter(new FileWriter("wdist.txt"));
    PrintWriter out5 = new PrintWriter(new FileWriter("nstrong.txt"));
    PrintWriter out6 = new PrintWriter(new FileWriter("avdev.txt"));
    PrintWriter out7 = new PrintWriter(new FileWriter("wbig.txt"));
    PrintWriter out8 = new PrintWriter(new FileWriter("n12.txt"));
    PrintWriter out9 = new PrintWriter(new FileWriter("clust.txt"));

/* Note on above output files. clust.txt outputs a cluster time course like that of Figs. 3A or 3B in the manuscript. In this multicolumn ASCII file the odd numbered columns are the time in days and the even numbered columns are the weight time courses for the 10 synapses. wdist.txt outputs the steady-state weight histogram from Fig. 2A, as well as the fitted log-normal distribution. nstrong.txt outputs the time course of Fig. 3C. deldist.txt outputs the steady-state delta W histogram from Fig. 4A as well as the fitted smooth distribution that lacks the sharp peak near 0. delhist.txt outputs the histogram from Fig. 4B, showing the mean of delta W plotted against W, as well as showing the curves for 1 standard deviation above and below the mean. */

	int rectime=1; // depends on aknt and recend

    	double recstart=0.0; // Time to start writing data

	double recend=60000; /* Time to end simulation. Time units of “imaging frames”, arbitrary but say once every 24 hrs. Then have to have another variable “tday” to print out time course in days.*/

	double delt = 1.0;
	double tday;

	int ltptime = 58000;

	int nstep=0;
	int n1=0;
	int n1and2=0;
	int n1thru2=0;

	int nwalk=10;
	int nclust=1000;

	double wsyn;
	double delwup=0.0;
	double delwdn=0.0;
	double totdel=0.0;

	double whi=-1000.0;
	double wlo=1000.0;
	int nbin = 80;
	double bnum=(double)(nbin);
	double winc;
	double chksum=0.0;
	double wdev=0.0;
	double wav=0.0;

	double dhi=-1000.0;
	double dlo=1000.0;
	double dinc;

	double sumint;
	double sumint2;

	int nbig=0;
	double pregen=0.1; // 0.1 ST
	double ratt;

	double wbas = 0.05; // 0.05 ST
	double fracmax = 0.05; // 0.05 ST
	double wmax = 20.0; // 20.0 ST
	double winit = 1.0; // 1.0 ST, strong init vals cause it's a cluster
	double wres = 0.7; // 0.7 ST. moderate but not strong W for random occasional “recreation” of synapses that have gone silent.
	double strth = 0.8; // 0.8 ST, threshold for “strong” synapse, below is “weak”
	double wkth = 0.08; // 0.08 ST, threshold for “weak” synapse. Set to wbas below this and make eligible to regenerate new synapse.
	double wdev1=0.045; // 0.045 ST Standard deviation for LTP Gaussian distribution, before bias correction.
	double wdev1c; // Standard dev for LTP Gaussian after correction.
	double wdev2=0.04; // 0.04 ST. Standard deviation for LTD Gaussian distribution.
	double upbias=0.80; // 0.80 ST, bias to make up LTP steps a little smaller. Goes with wdevc.
	double upset=0.88; // 0.88 ST. Goes along with upbias.

	double wav1; // mean of LTP Gaussian
	double wav2; // mean of LTD Gaussian
	double av1fact = 4.0; // SD 4.0, ratio of mean to SD of LTP Gaussian.
	double av2fact = 4.0; // SD 4.0, ratio for LTD Gaussian.

	double volat;
	double vfact=4.0; // These parameters make the smaller spines more volatile than the larger ones.

	double vset=0.4;
	double lowval=0.2;

	double adfact;
	int nset=6; // 6 ST
	double ampfact = ((double)(nset))/((double)(nwalk-nset));

	double r1, r2, r3, fac1, fac2cos, fac2sin, z1, z2;

	int kntcl = 0;

	r1=Math.random();
	r1=Math.random();

	double fcent=0.0;
	double fdev=1.0;
	double famp=300.0;
	double bcent;
	double efact;

	double dbcent;
	double defact;
	double dfcent=0.0;
	double dfdev=0.07;
	double dfamp=52.0;

	double rnum1;
	double rnum2;
	double ppi = 3.14159265;

	double trx;
	double time; 

    int i, k, j, iout, iknt=1; 

	double[] wvals = new double[nwalk+3];
	double[] wnorm = new double[nbin+2];
	double[] dnorm = new double[nbin+2];
	double[] whist = new double[nbin+2];
	double[] dhist = new double[nbin+2];
	double[] wbig = new double[50000];

	int[] ivals1 = new int[nwalk+3];
	int[] ivals2 = new int[nwalk+3];
	int[] ivals3 = new int[nwalk+3];
	int[] ibig3 = new int[50000];
	int[] ibig2 = new int[50000];
	int[] ibig1 = new int[50000];

	double[] avdel = new double[202];
	double[] avknt = new double[202];
	double[] semdel = new double[202];
	double[] wcent = new double[202];
	double tmp = 0.0;

	double[] delvals = new double[nwalk+3];
	double[] lastdel = new double[50000];

	double[] wav5 = new double[5000];
	double[] wsum5 = new double[5000];
	double[] wsdev5 = new double[5000];
	double[] dwav5 = new double[5000];
	double[] dwsum5 = new double[5000];
	double[] dwsdev5 = new double[5000];

        i=0;
        do { 
		wav5[i]=0.0;
		wsum5[i]=0.0;
		wsdev5[i]=0.0;
		dwav5[i]=0.0;
		dwsum5[i]=0.0;
		dwsdev5[i]=0.0;
	   i++;
         } while (i <= 4990);

        i=0;
        do { 
		wbig[i]=wbas;
		ibig1[i]=0;
		ibig2[i]=0;
		ibig3[i]=0;
		lastdel[i]=0.01;
	   i++;
         } while (i <= 49990);

do {

	kntcl = kntcl+1;

        i=1;
        do { 
		ivals1[i]=0;
		ivals2[i]=0;
		ivals3[i]=0;
	   i++;
         } while (i <= nwalk+2);

	wvals[1]=0.0;
	wvals[nwalk+2]=0.0;
        i=2;
        do { 
		wvals[i]=winit;
	   i++;
         } while (i <= nwalk+1);

	delvals[1]=0.0;
	delvals[nwalk+2]=0.0;
        i=2;
        do { 
		delvals[i]=0.01;
	   i++;
         } while (i <= nwalk+1);

        i=1;
        do { 
		wnorm[i]=0.0;
		whist[i]=0.0;
	   i++;
         } while (i <= nbin+1);

        do { 
		avdel[i]=0.0;
		avknt[i]=0.0;
		semdel[i]=0.0;
	   i++;
         } while (i <= 200);

	time = 0.0;
	nstep = 0;

      do {  

	time = time + delt;

	    iout=2;
	    do { 

	wsyn = wvals[iout];

		r1=Math.random();
		r2=Math.random();
		fac1 = -2.0*Math.log(r1);
		fac1 = Math.sqrt(fac1);
		fac2cos = Math.cos(-2.0*ppi*r2);
		fac2sin = Math.sin(-2.0*ppi*r2);
	adfact = ampfact*((double)(nwalk-nbig))*(upset-upbias)/((double)(nset));

		wdev1c = wdev1*(upbias+adfact);
		wav1 = av1fact*wdev1c;
		z1 = fac1*fac2cos*wdev1c + wav1;
		if (z1 < 0.0) {z1 = 0.0;}

		wav2 = av2fact*wdev2;
		z2 = fac1*fac2sin*wdev2 + wav2;
		if (z2 < 0.0) {z2 = 0.0;}

	volat = (vfact-(vfact-lowval)*(wsyn/(wsyn+vset)))*wsyn;

	delwup = z1*volat*(1.0-fracmax*wsyn/(wsyn+wmax)); 

	delwdn = z2*volat;

	totdel = delwup-delwdn;

	if (wsyn == wbas) {totdel = 0.0;}

	wsyn = wsyn+totdel;

	if (wsyn < wkth) {wsyn = wbas;}

	wvals[iout] = wsyn;

	delvals[iout] = delwup-delwdn;

	ivals1[iout]=0;

	if (wsyn > strth)
	{
		ivals1[iout]=1;
	}

	   iout++;
         } while (iout <= nwalk+1);

	  nbig=0;
        k=2;
        do { 
		if (wvals[k] > strth) 
			{
			nbig=nbig+1;
			}
	   k++;
         } while (k <= nwalk+1);

	    iout=2;
	    do { 

		wsyn = wvals[iout];

	ratt = ((double)(nbig))/((double)(nwalk));

	r3=Math.random();
	// r3 = 1000.0;
if ((wsyn == wbas) && (r3 < pregen*ratt) && (wvals[iout-1] > strth)) {wsyn = wres;}
if ((wsyn == wbas) && (r3 < pregen*ratt) && (wvals[iout+1] > strth)) {wsyn = wres;}

		wvals[iout] = wsyn;

	   iout++;
         } while (iout <= nwalk+1);


	  nbig=0;
        k=2;
        do { 
		if (wvals[k] > strth) 
			{
			nbig=nbig+1;
			}
	   k++;
         } while (k <= nwalk+1);

/* Following is to reset synapses to a fixed value, 5 high, 5 low, mimicking imposed LTP and LTD to give a “memory pattern”. Comment this out if you want to output steady state synaptic weight distribution without resetting. */
/* if (nstep == 50300)
 	{
	wvals[2]=5.0;
	wvals[3]=5.0;
	wvals[4]=5.0;
	wvals[5]=5.0;
	wvals[6]=5.0;
	wvals[7]=0.5;
	wvals[8]=0.5;
	wvals[9]=0.5;
	wvals[10]=0.5;
	wvals[11]=0.5;
	}
*/
if ((nstep > ltptime) && (nstep < ((int)(recend))) && (iknt == 50))
 	{
	tday = time+0.1-((double)(ltptime));
out9.println(tday + "\t" + wvals[2] + "\t" + tday + "\t" + wvals[3] + "\t" + tday + "\t" + wvals[4] + "\t" + tday + "\t" + wvals[5] + "\t" + tday + "\t" + wvals[6] + "\t" + tday + "\t" + wvals[7] + "\t" + tday + "\t" + wvals[8] + "\t" + tday + "\t" + wvals[9] + "\t" + tday + "\t" + wvals[10] + "\t" + tday + "\t" + wvals[11]);
	}

if ((nstep > (ltptime-4000)) && (nstep < ltptime) && (iknt == 50))
 	{
tday = (time-48000.0)*24.0/24.0;
out5.println(tday + "\t" + nbig);
	}


/* 
k=1;
if ((nstep > ltptime) && (nstep < ((int)(recend))))
 	{
k=(nstep-ltptime);
dwav5[k] = (wvals[2]+wvals[3]+wvals[4]+wvals[5]+wvals[6])/5.0;
	}
k=1;
*/

		nstep=nstep+1;

           } while (nstep < ((int)(recend)));

        k=1;
        do { 
		wbig[k+(iknt-1)*nwalk]=wvals[k+1];
		lastdel[k+(iknt-1)*nwalk]=delvals[k+1];
		ibig1[k+(iknt-1)*nwalk]=ivals1[k+1];
		ibig2[k+(iknt-1)*nwalk]=ivals2[k+1];
		ibig3[k+(iknt-1)*nwalk]=ivals3[k+1];
	   k++;
         } while (k <= nwalk);

	  /*  k=1;
	   do {
		wav5[k] = wav5[k] + dwav5[k]/((double)(nclust));
		k++;
		} while (k <= (recend-50001));
	*/ 
	   iknt++;
         } while (iknt <= nclust);

/* In following loop, log statement is to take log of W's to show lognormal distribution of W's. If want to show tailed distribution of raw W's instead, comment out the log statement. */      
        i=1;
        do { 
		wbig[i] = Math.log(wbig[i]);
		wav = wav+wbig[i]/((double)(nwalk*iknt));
		i++;
          } while (i <= nwalk*(iknt-1));

        i=1;
        do { 
		wdev = wdev + (wbig[i]-wav)*(wbig[i]-wav);
		i++;
          } while (i <= nwalk*(iknt-1));

		wdev=wdev/((double)(nwalk*(iknt-1)));
		wdev=Math.sqrt(wdev);

        i=1;
        do { 
		if (wbig[i] > whi) {whi=wbig[i];}
		if (wbig[i] < wlo) {wlo=wbig[i];}
		i++;
          } while (i <= nwalk*(iknt-1));

		winc = (whi-wlo)/bnum;

        i=1;
        do { 
        j=1;
        do { 
		if ((wbig[i] > (0.999999*wlo+(double)(j-1)*winc)) && (wbig[i] < (1.000001*wlo+(double)(j)*winc))) {whist[j]++;}
		j++;
          } while (j <= nbin);
		i++;
          } while (i <= nwalk*(iknt-1));

        i=1;
        do { 
		if (lastdel[i] > dhi) {dhi=lastdel[i];}
		if (lastdel[i] < dlo) {dlo=lastdel[i];}
		i++;
          } while (i <= nwalk*(iknt-1));

		dinc = (dhi-dlo)/bnum;

        i=1;
        do { 
        j=1;
        do { 
		if ((lastdel[i] > (0.999999*dlo+(double)(j-1)*dinc)) && (lastdel[i] < (1.000001*dlo+(double)(j)*dinc))) {dhist[j]++;}
		j++;
          } while (j <= nbin);
		i++;
          } while (i <= nwalk*(iknt-1));

        i=1;
        do { 
		bcent = wlo + (0.5+(double)(i-1))*winc;
		efact = -0.5*(fcent-bcent)*(fcent-bcent)/(fdev*fdev);
		wnorm[i] = (famp/fdev)*(Math.exp(efact));
		i++;
          } while (i <= nbin);

        i=1;
        do { 
		dbcent = dlo + (0.5+(double)(i-1))*dinc;
		defact = -0.5*(dfcent-dbcent)*(dfcent-dbcent)/(dfdev*dfdev);
		dnorm[i] = (dfamp/dfdev)*(Math.exp(defact));
		i++;
          } while (i <= nbin);

        i=1;
        do { 
		bcent = wlo + (0.5+(double)(i-1))*winc;
		dbcent = dlo + (0.5+(double)(i-1))*dinc;
		out3.println(dbcent + "\t" + dhist[i] + "\t" + dnorm[i]);
		out4.println(bcent + "\t" + whist[i] + "\t" + wnorm[i]);
		i++;
          } while (i <= nbin);

	out6.println(wav + "\t" + wdev + "\t" + whi + "\t" + wlo + "\t" + dhi + "\t" + dlo);

        i=1;
        do { 
		out7.println(i + "\t" + wbig[i]);
		i++;
          } while (i <= nwalk*(iknt-1));

        i=0;
        do { 
		if (ibig1[i] == 1) {n1=n1+1;}
		if ((ibig1[i] == 1) && (ibig2[i] == 1)) {n1and2=n1and2+1;}
		if (ibig3[i] == 1) {n1thru2=n1thru2+1;}
	   i++;
         } while (i <= 49000);

	out8.println(n1 + "\t" + n1and2 + "\t" + n1thru2);

        i=1;
	  j=1;
	  k=1;
        do { 
		j=i;
		k=i;
	    do {
		  if (wbig[j] > wbig[k])
		  {
		  j = k;
		  }
		  k++;
             } while (k <= nwalk*(iknt-1));
		 tmp = wbig[i];
		 wbig[i] = wbig[j];
		 wbig[j] = tmp;
		 tmp = lastdel[i];
		 lastdel[i] = lastdel[j];
		 lastdel[j] = tmp;
		i++;
          } while (i <= nwalk*(iknt-1));

        i=1;
        do { 
        j=1;
        do { 
		k=j+(49*(i-1));
		avdel[i]=avdel[i]+Math.abs(lastdel[k]/49.0);
		wcent[i]=wcent[i]+wbig[k]/49.0;
		j++;
          } while (j <= 49);
		i++;
          } while (i <= 200);

        i=1;
        do { 
        j=1;
        do { 
		k=j+(49*(i-1));
	semdel[i] = semdel[i] + (lastdel[k]-avdel[i])*(lastdel[k]-avdel[i]);
		j++;
          } while (j <= 49);
		i++;
          } while (i <= 200);

        i=1;
        do { 
		semdel[i] = semdel[i]/49.0;
		semdel[i] = Math.sqrt(semdel[i]);
		semdel[i] = semdel[i]/(Math.sqrt(49.0));
		i++;
          } while (i <= 200);

        i=1;
        do { 
		out1.println(wbig[i] + "\t" + Math.abs(lastdel[i]));
		i++;
          } while (i <= nwalk*(iknt-1));

        i=1;
        do { 
		if (wcent[i] > wbas)
		{
		out2.println(wcent[i] + "\t" + (avdel[i]-semdel[i]) + "\t" + avdel[i] + "\t" + (avdel[i]+semdel[i]));
		}
		i++;
          } while (i <= 200);

      out1.close();
      out2.close();
      out3.close();
      out4.close();
//      out5.close();
      out6.close();
      out7.close();
      out8.close();
      out9.close();

       }
}
